# Multiple Evaluations of the Spatial and Temporal Characteristics of Surface Water Quality in the Typical Area of the Yangtze River Delta of China Using the Water Quality Index and Multivariate Statistical Analysis: A Case Study in Shengzhou City

**DOI:** 10.3390/ijerph20042883

**Published:** 2023-02-07

**Authors:** Yang Liu, Lijuan Li

**Affiliations:** 1 Key Laboratory of Water Cycle and Related Land Surface Processes, Institute of Geographic Sciences and Natural Resources Research, Chinese Academy of Sciences, Beijing 100101, China; 2 College of Resources and Environment, University of Chinese Academy of Sciences, Beijing 100049, China

**Keywords:** Yangtze River Delta, water quality, water quality index, multivariate statistical analysis

## Abstract

Surface water assessments are of critical importance for balancing economic development with the ecological environment in rapidly developing regions. In this research, Shengzhou City, a typical town in the Yangtze River Delta region of China, was chosen to conduct a surface water quality study. As a region with a well-developed water system, monthly water quality monitoring data from eight sampling sites on the major tributaries and the mainstream were selected for six consecutive years from 2013 to 2018, containing seven important water quality indicators (pH, DO, COD_Mn_, COD_Cr_, BOD, NH_4_^+^-N, and TP). The comprehensive evaluation method based on the water quality index (WQI) and multivariate statistical analysis methods of cluster analysis (CA) and principal component analysis (PCA) were applied to explore the spatial and temporal changes of water quality in Shengzhou City. The main findings are as follows: (1) spatially, for three main tributaries, Xinchang River had the worst water quality, followed by Changle River, while Huangze River had the best. The water quality of the tributaries had higher volatility than the mainstream. (2) The sampling sites with similar locations had similar water quality characteristics. (3) Seasonally, for the four indicators of DO, COD_Mn_, COD_Cr_, and BOD, the water quality was better in the dry season while, for NH_4_^+^-N and TP, water quality was better in the wet season. The low WQI points were more likely to appear in the wet season. (4) The results of WQI assessment showed an improving trend in water quality. (5) Nitrogenous substances and organic matter were the key pollutants in this area. The research results prove that water quality evaluation methods and multivariate statistical methods are effective for the study of regional surface water quality.

## 1. Introduction

Water quantity and water quality are of vital importance to human society. In recent years with economic and social development, point source pollution, including industrial wastewater and domestic sewage, and non-point source pollution mainly caused by agricultural activities, have made the water environment fragile. Global freshwater use has increased by a factor of six over the past 100 years. Other than that, globally an estimated 80% of all industrial and municipal wastewater is released into the environment without any prior treatment. Managing excess nutrients in agricultural runoff is also one of the most prevalent water quality-related challenges. Hundreds of chemicals are negatively impacting water quality and ecosystems [1]. Clearly, water resources management is a global issue. Many regions in the world are suffering from the economic impacts of water-related problems [2,3]. However, water quality is affected by many natural factors and anthropogenic factors [4,5], presenting complex challenges to water resource management [6]. Various surface water properties should be evaluated when developing water management plans [7]. Therefore, water quality assessment is an important part of water resources management, and many assessment methods have emerged.

Water resource problems are common throughout China [2]. According to the second national survey of pollution sources in China, the amount of total water pollutant discharge was huge in 2017, including 21,439,800 tons of chemical oxygen demand, 963,400 tons of ammonia nitrogen, 3,041,400 tons of total nitrogen, and 315,400 tons of total phosphorus. The Yangtze River Delta is one of the regions where the overall environment has changed the most during the process of urbanization in China. The dense water network and abundant water resources have contributed to the strong economic strength of the Yangtze River Delta, while the high-speed process of urbanization and industrialization has destroyed the water environment [8,9,10]. The contradiction between the status of resources and environment, and the development of society and economy is gradually emerging, making the Yangtze River Delta an ecologically fragile region. As a result, it is a matter of great urgency to understand the reasons for surface water quality change [11].

Analysis of spatial and temporal characteristics of surface water quality usually involves biological, chemical, physical, and radiological parameters monitored at multiple sampling times (temporal variation) and sampling sites (spatial variation). It is very complicated due to a large amount of data, the strong correlation between parameters, and the different units of parameters [12]. Therefore, in water quality research, simplifying data information and reducing subjectivity and uncertainty of evaluation have been the focus of scholars’ work.

Water quality evaluation is the basis of watershed management. At present, the main methods of water quality evaluation include both single-factor evaluation and comprehensive evaluation methods [13]. Single-factor evaluation methods are traditional, comparing the parameter values determined experimentally with the local standard values individually. Comprehensive evaluation methods can reflect the overall impact of multiple pollutants on water quality by summarizing and counting single pollutant values mathematically. The water quality index (WQI) is a typical comprehensive evaluation method, initially developed by Horton [14] in the 1960s, and constructed by aggregating the physical and chemical factors of the water body to a single value [5], which avoids the bias of single-factor evaluation making identification of the overall variation trend and interregional comparison possible. This method has been widely used in water environment research at home and abroad, providing an efficient and understandable approach for policymakers and watershed managers. A range of national and international organizations have proposed various WQI forms, such as the National Sanitation Foundation Water Quality Index (NSF WQI), the British Columbia Water Quality Index (BC WQI), and the Canadian Council of Ministers of the Environment Water Quality Index (CCME WQI). Many indices are based on the NSFWQI [7], the most common composite index for the global classification of surface water quality [15].

Multivariate statistical methods, such as cluster analysis (CA) and principal component analysis (PCA), have been widely applied in the interpretation of complex monitoring water quality data and assessing spatiotemporal dynamics of water quality [16,17], with the advantages of simplifying data structure and extracting potential information. CA is applied to classify water samples on the basis of the similarity of water quality in order to excavate the spatiotemporal characteristics of water quality [18] and divide the degree of water pollution in space. PCA is adept at data reduction, which can help extract the pattern and most meaningful variables that cause variations to water quality [19] with minimum loss of original information. This method has gradually become the most commonly used method in water quality research.

Combining water quality evaluation methods with multivariate statistical methods, a more complete water quality research system is formed. The former aims to judge the overall water quality situation and trends, while the latter is intended for deep exploration of the spatial and temporal characteristics of water quality data and key indicators.

To explore the surface water quality characteristics of cities under the background of high-speed urbanization in the Yangtze River Delta, this research selected a representative city of the Yangtze River Delta, Shengzhou City, as the research area. This choice was based on the following three aspects:

(1) Geographical location: Shengzhou belongs to the “One-hour economic circle” of Zhejiang Province. It will become an important transportation hub in eastern Zhejiang with strong development potential and large water demand.
(2) Water system: Shengzhou has a well-developed water network. The Cao’e River passes through the territory, and is an important industrial and agricultural water source.
(3) Economic development: The rapid industrial development of Shengzhou brings a great risk of water pollution. The development of the manufacturing industry has caused point-source pollution, while the high level of agriculture has caused non-point source pollution.

Water quality management has always been an important environmental protection topic in Shengzhou. First, the textile industry produces a large amount of industrial effluent discharge. Second, agricultural non-point source pollution has a great impact on the water environment due to the important status of planting and farming in Shengzhou. Precipitation and runoff conditions increase the potential for pollution to occur. In addition, transboundary pollution problems have made water quality management more difficult.

In this study, the water quality of Shengzhou was analyzed by combining WQI with CA and PCA on the basis of the monthly surface water quality monitoring data of sampling sites from 2013 to 2018. First, the spatial and seasonal difference of individual water quality indicators throughout the study period is described, and a correlation analysis of the indicators performed. Second, as a comprehensive evaluation method, WQI was utilized to condense a set of data into a value to achieve a visual comparison of water quality between sampling sites and periods. Lastly, combined with multivariate statistical analysis methods, more effective information can be mined from the water quality data. On the basis of CA, the similarity of water quality data at sampling sites is calculated, and the spatial characteristics of water quality in the study area can be obtained. According to the PCA results of water quality data with different organizational forms, the key water quality indicators can be determined from multiple perspectives of the region—sampling sites—monitoring years to identify the main pollutants.

## 2. Materials and Methods

### 2.1. Study Area

**Figure 1:**
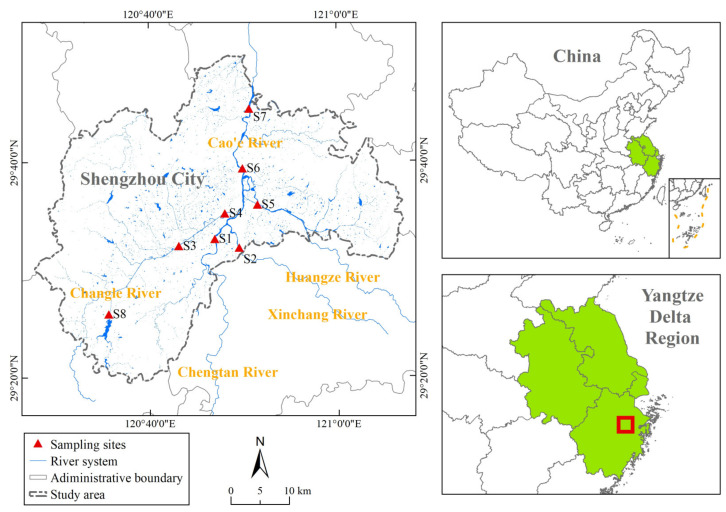
Location of the study area and sampling sites.

Shengzhou City (29°19′45″–29°49′55″N, 120°27′23″–121°06′55″E) is situated in Zhejiang Province, in the Yangtze River Delta of China. The location of the study area is shown in Figure 1. The city is 64.1 km long from east to west and 55.4 km wide from north to south, with a total area of 1789 km^2^. Shengzhou is surrounded by mountains, sloping from southwest to northeast. The main terrain type is the basin and low mountains, with the area of hills and mountains accounting for 77%. It is a typical mountainous city, with a 7:1:2 area ratio of mountains, waters, and fields.

From a hydrological perspective, the research area is located in the upper reaches of the Cao’e River and crossed by the river from south to north, which is the largest tributary of the Qiantang River. The total length of Cao’e River is 193 km, and the length within Shengzhou is 32.2 km, with an average annual flow of 56 m^3^/s. Chengtan River is regarded as the source of the Cao’e River, with a length of 91 km and an average annual flow of 20.8 m^3^/s. The developed river system in the territory is typically fan-shaped, with Chengtan River and three important tributaries, Changle River (length: 78 km, flow: 19.1 m^3^/s), Xinchang River (length: 67 km, flow: 9.9 m^3^/s), and Huangze River (length: 71 km, flow: 12.4 m^3^/s), successively converging in the territory of Shengzhou. The upper reaches of Cao’e River are surrounded by mountains and situated in flashy rivers.

Climatically, the area is in the subtropical monsoon climate zone with prominent monsoon features; four distinct seasons, mild climate, and abundant precipitation. The annual average temperature is 16.4 °C, the sunshine duration is 1988 h, and the frost-free period is 235 days. The annual average precipitation is 1447 mm, and precipitation occurs on 140–180 days. The precipitation distribution is uneven throughout the year. The wet season begins in April, and there are two obvious rainy seasons throughout the year, the plum rain season (from June to July) and the typhoon rain season (from August to September), when the rainfall is relatively concentrated and heavy. Topographic and climatic conditions are prone to serious flood disasters and soil erosion.

Shengzhou was one of the first batches of national coastal counties (cities) with economic openness, and its location and transportation advantages have brought development opportunities. The manufacturing industry has obvious characteristics, mainly the electrical processing and textile industries. Modern agriculture is booming, and the planting area of high-quality agricultural products is gradually expanding. It is an important tea production base with a planting area of 11,867 hm^2^. In addition, it is a national pig breeding base. The livestock and poultry breeding industry of Shengzhou has a long history and a high degree of professionalism. Correspondingly, the main sources of water pollution in Shengzhou are point-source pollution caused by domestic sewage and industrial wastewater, and non-point source pollution caused by farmland soil erosion and livestock and poultry breeding sewage.

### 2.2. Sampling Strategy and Analytical Procedure

Water samples were collected once a month from 2013 to 2018 at different sampling sites covering the mainstream and important tributaries by the Shengzhou Ecology and Environment Bureau. To analyze seasonal changes in water quality, each year was divided into wet and dry seasons. The wet season was taken as April to September, while the dry seasons were taken as January to March and October to December.

Eight sampling sites were picked to represent the water quality of Shengzhou according to the location and data integrity; the locations of the sites are shown in Figure 1. Site 1 (S1) was located in Chengtan River, representing the water quality of Chengtan River and Shengzhou’s inbound water quality. For the tributaries, Site 2 (S2) was distributed along Xinchang River. Site 3 (S3) and Site 4 (S4) were distributed along Changle River. Changle River is the longest tributary in Shengzhou with the largest watershed area; accordingly, two sites were chosen. Site 5 (S5) was distributed along Huangze River. Site 6 (S6) and Site 7 (S7) were located in the mainstream of Cao’e River. Site 6 was located downstream of the confluence of the important tributaries, the beginning of the mainstream of Cao’e River, where the water flow becomes larger. Site 7 was near the border of Shengzhou, regarded as the outbound water quality. To measure the state of the important drinking water source, Site 8 (S8) of Nanshan Reservoir was taken into account.

Seven parameters were selected to construct the system for the analyses. The potential of hydrogen (pH, unitless) and dissolved oxygen (DO, mg/L) were directly determined in situ using a multiparameter water quality monitoring meter. Water samples from each site were collected in PE bottles and analyzed ex situ for laboratory testing. The permanganate index (COD_Mn_, mg/L) was determined using the permanganate method, chemical oxygen demand (COD_Cr_, mg/L) ascertained using the bichromate method, and biochemical oxygen demand (BOD, mg/L) using the dilution and inoculation method. Verification of ammonium nitrogen (NH_4_^+^-N, mg/L) was carried out using Nessler’s reagent colorimetric method. Total phosphorus (TP, mg/L) was determined using the ammonium molybdate spectrophotometric method. Pretreatment and mensuration for these parameters were all conducted following the “Technical Specifications for Environmental Monitoring” issued by the State Environmental Protection Administration. Among them, pH is the acid–base state of the water body. DO was used to reflect the oxidation conditions and self-purification capacity of the water body. COD_Mn_, COD_Cr_, and BOD were all used to indicate the amounts of reducing substances in water, wherein the difference lies in the oxidant. The ratio of BOD to COD_Cr_ is an indication of how difficult it is for organic pollutants in water to be decomposed by microorganisms, which are more harmful to the environment. NH_4_^+^-N and TP were used to reflect the nutritional status of the water body.

For individual missing data, the annual average value was used for replacement. When the value was lower than the detection limit concentration of the detection method, analysis was performed according to the concentration value of half of the minimum detection limit.

In summary, this research selected seven water quality parameters at eight sampling sites from 2013 to 2018 (the data of S2 were from 2013 to 2016, and the data of S3 were monitored once every two months in 2017), with a total of 546 pieces of water quality data.

### 2.3. Water Quality Index (WQI)

The water quality index (WQI) is a widely used comprehensive water quality factor evaluation method, which has developed various forms in practical applications. WQI is generally developed in a four-step process: (1) parameter selection, (2) indicator scoring, (3) weight assignment, and (4) aggregation of scores and weights in a single value [15].

This study adopted the calculation formula refined and developed by Pesce and Wunderlin [20]:

(1) $$\mathrm{WQI}=\frac{\sum_{i=1}^{n}C_{i}\times P_{i}}{\sum_{i=1}^{n}P_{i}},$$

where WQI is the water quality index, *C_i_* is the standardized score of water quality parameter *i,* and *P_i_* is the weight of water quality parameter *i*; the minimum value of *P_i_* is 1 and the maximum value is 4 [21].

This method is based on the real situation of river water quality. The score and weight of each indicator are determined on the basis of a large number of previous research results and the degree of harm to water health. The values of *C_i_* and *P_i_* in this study are listed in Table 1 [20,21,22,23].

According to the WQI value, water quality can be classified into five levels as follows: excellent [90–100], good [70–90), medium [50–70), poor [25–50), very poor [0–25). The monthly WQI value was calculated by taking the monthly average value of water quality parameters, the seasonal WQI value was calculated by taking the seasonal average value, and the annual WQI value was calculated by taking the annual average value.

### 2.4. Cluster Analysis (CA) and Principal Component Analysis (PCA)

Cluster analysis (CA), which classifies samples according to the similarity of data or attributes, was used to explore the spatial heterogeneity of water quality in the watershed. The core of the method lies in the measure of distance or similarity between samples. In this study, the hierarchical clustering analysis (HCA) was adopted with the Euclidean distance to measure the distance between samples, and the sum of squares of deviations algorithm (Ward method) was used to hierarchically cluster the standardized dataset. The Ward method is the most commonly used clustering algorithm. The calculation results in the individuals within each cluster having strong similarities, while the heterogeneity among the clusters is strong.

The basic process is as follows:

- Data preprocessing: Standardize the original data to eliminate dimensional effects.
- Measurement of distance or similarity: Adopt Euclidean distance to measure the distance between samples.
- Determination of clustering method: Use the Ward method to analyze the spatial similarity of the samples, so as to cluster the similar samples together.

Principal component analysis (PCA) was used to identify the main factors that cause spatial differences in water quality. The basic idea of PCA is dimensionality reduction. First, multidimensional variables are incorporated into the same system. Second, the system is simplified by creating new variables under the principle of least data loss. Lastly, key factors are screened and further quantitative analysis is performed. The application of this method in water quality evaluation is to introduce complicated water quality data into the same system, explore key pollution indicators by simplifying the data structure, and reflect the pollution status of water bodies through the comprehensive score of principal components.

The basic process is as follows:

- Data preprocessing: Standardize the original data to eliminate dimensional effects.
- Statistical test of applicability: Calculate the correlation coefficient matrix between every two indicators.
- Determination of principal components: Calculate eigenvalues and contribution rates to determine principal components.
- Quantitative analysis: Obtain the principal component loading matrix to determine the main factors.

The analyses and plots were performed using Microsoft Excel 2019 (Microsoft Corporation, Redmond, WA, USA), MATLAB version R2019b (MathWorks, Natick, MA, USA), and Origin 2021 software (OriginLab, Northampton, MA, USA).

## 3. Results and Discussions

### 3.1. Water Quality Characteristics of Shengzhou City

The spatiotemporal characteristics of the water quality of the whole basin and each sampling site from 2013 to 2018 were obtained by analyzing the 546 sets of water quality data. Due to a large amount of data, only the key statistics (min, max, mean, and standard deviation (SD)) of water quality parameters are given in Table 2.

During the study period, the pH of surface water in Shengzhou City ranged from 6.110 to 8.96, with an average level of 7.459, showing a weak alkalinity overall. The DO range was 3.120–15.500 mg/L with an average value of 8.737 mg/L. COD_Mn_ ranged from 0.080 to 7.430 mg/L, with an average of 2.693 mg/L. COD_Cr_ ranged from 2.000 to 28.000 mg/L, with an average value of 11.273 mg/L. BOD ranged from 0.250 to 6.510 mg/L, with an average of 2.315 mg/L. NH_4_^+^-N ranged from 0.007 to 1.720 mg/L, with an average value of 0.374 mg/L. TP ranged from 0.003 to 0.289 mg/L, with an average of 0.077 mg/L.

Among these water quality records, the minimum values of pH and DO appeared in S6. The maximum values of COD_Mn_, BOD, and NH_4_^+^-N all appeared in S2, while the maximum values of COD_Cr_ and TP appeared in S7. According to China’s Surface Water Environment Quality Standard, the minimum DO reached the Class IV water quality standard, as did the maximum values of COD_Mn_, COD_Cr_, and TP in the study area. The maximum values of BOD and NH_4_^+^-N even met the Class V water quality standard, indicating poor water quality in the research area.

#### 3.1.1. Spatial Differences

There were obvious spatial differences in water quality among sampling sites. Table 3 shows the mean and standard deviation of the water quality parameters of each sampling site from 2013 to 2018. The main conclusions are drawn below.

Among all the sites, the mean COD_Cr_, BOD, NH_4_^+^-N, and TP of S2 were the highest with the largest variation of NH_4_^+^-N and TP. This phenomenon represents that the water entering Shengzhou from Xinchang River was poor. This is because there are many pharmaceutical and chemical companies in the upper reaches of Xinchang River which discharged substantial industrial wastewater. Transboundary pollution is a common water environment problem in the Yangtze River Delta region.

S4 had the highest mean DO and COD_Mn_, as did the amplitude of variation of the two indicators. In addition, the COD_Cr_ and TP levels of S4 are worthy of attention. Changle River is famous for its developed agriculture. As a result, agricultural activities and rural life have caused non-point source pollution, bringing substantial organic pollutants and nitrogen and phosphorus pollutants into the water body. Meanwhile, a high DO level indicated that the water body had strong self-purification capacity.

The average DO of S7 was the lowest, while the COD_Cr_ and BOD of S7 changed the most during the study period. Its COD_Mn_ ranked second among all stations. As a sampling site downstream of the research area on the mainstream of Cao’e River, S7 received the pollutants along the way and from the inflow of tributaries, leading to poor self-purification capacity.

The average COD_Mn_, COD_Cr_, BOD, NH_4_^+^-N, and TP of S8 were the lowest, indicating that the water quality of the reservoir was the best. In general, the water quality of S2, S4, S6, and S7 deserves special attention.

BOD/COD indicates the biodegradability of water. The multiyear average BOD/COD values for each sampling site ranged from 0.162 to 0.263, which is lower than 0.3, indicating that the water environment in this area is difficult to biodegrade.

#### 3.1.2. Seasonal Differences

The water quality of rivers is directly affected by precipitation and runoff. The research area has distinct dry and wet seasons with concentrated precipitation, leading to obvious seasonal differences in water quality indicators.

There is less precipitation in the dry season, when the water body is mainly polluted by domestic water and industrial wastewater discharge. During the wet season, on the one hand, abundant rainfall could enlarge the risk of agricultural non-point source pollution. On the other hand, an increase in runoff accelerates the water cycle, and the dilution effect and self-purification capacity of water body are strong. Temperature changes, biological growth stages, and human activity characteristics also affect water quality, along with hydrologic changes.

**Figure 2:**
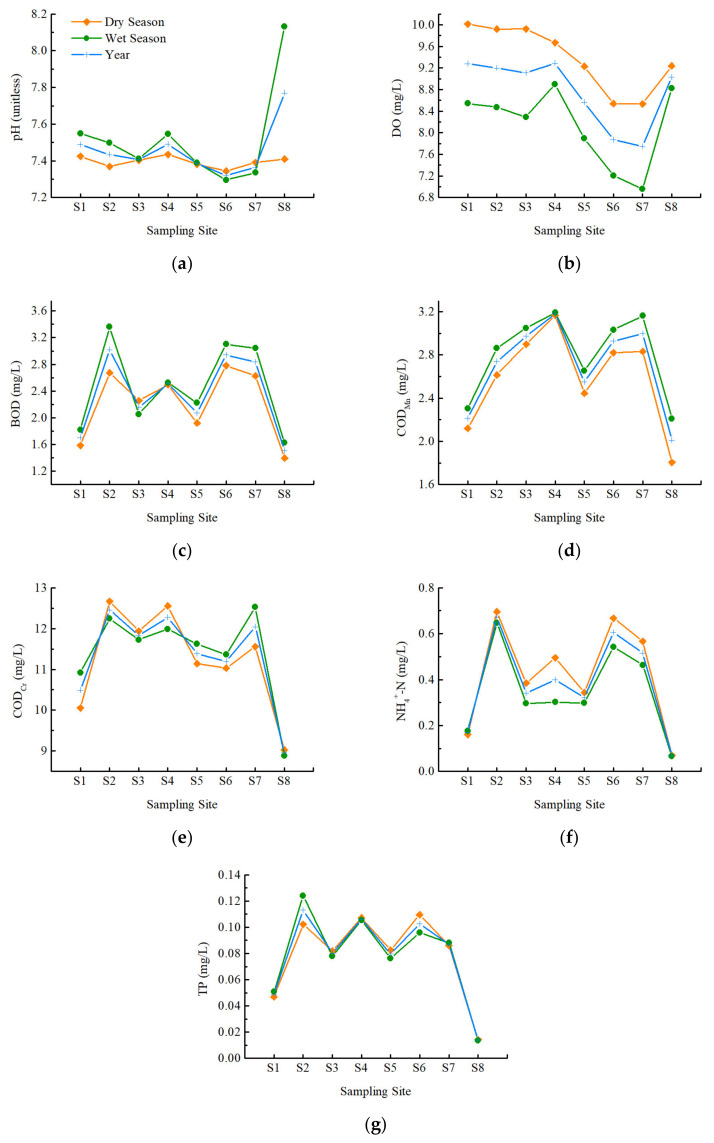
Water quality levels according to years, as well as dry and wet seasons, in each sampling site. (**a**). pH; (**b**). DO; (**c**). BOD; (**d**) COD_Mn_; (**e**) COD_Cr_; (**f**) NH_4_^+^-N; (**g**)TP.

According to the regional precipitation characteristics, each year was divided into dry seasons (January to March, October to December) and a wet season (April to September). In this study, the water quality indicators of each sampling site were analyzed separately according to different periods to obtain the multiyear average and multiyear seasonal average. Figure 2 shows the seasonal difference in water quality.

The pH values of the dry and wet seasons were both within the appropriate range. Except for S6 and S7, the pH values of the remaining sites in the wet season were higher than those in the dry season, indicating that the water quality was more alkaline in the wet season. There was a slight seasonal difference in the pH of S3 and S5, whereas there was a significant seasonal difference in S8.

The DO of each sampling site was higher in the dry season than in the wet season. The value of DO is influenced by many factors, such as temperature, pressure, microbial population, biological processes, dissolved salt density, sampling time [24], and human activity. The high temperature in the wet season leads to a decrease in the solubility of oxygen in water, while the high-temperature conditions accelerate the oxygen consumption process in the water body. DO increases in the cold season because low temperature increases the solubility of oxygen, and organisms reduce activities that require oxygen consumption.

The COD_Mn_ of each sampling site was higher in the wet season than in the dry season. For BOD, the wet season values were higher at all sites except S3. Both COD_Mn_ and BOD of S4 had little difference between dry and wet seasons. For COD_Cr_, sites with higher values in the wet season were S1, S5, S6, and S7; the opposite trend was seen at sites S2, S3, S4, and S8. The content of organic matter in the river water body was higher in the wet season, mostly related to the increase in primary production in the river. The wastewater discharged from the textile industry is mainly organic pollution, which would cause an increase in the relevant indicators. In addition, precipitation in the wet season can lead to a large number of agricultural non-point source organic pollutants and the overflow of the sewage pipe network entering the water body in a short time.

For NH_4_^+^-N, the sites showed that the dry season led to higher values than in the wet season, except for S1. There was little difference between dry and wet seasons in S8. For TP, sites with higher values in the wet season were S1, S2, and S7; the opposite trend was seen at sites S3, S4, S5, S6, and S8. There was little difference between dry and wet seasons in S4 and S8. The main source of pollution in the dry season is point-source pollution, and domestic sewage often contains substantial nitrogen and phosphorus pollutants. The rare precipitation in dry season leads to poor water quality adjustment capacity and a low dilution effect on pollutants. In addition, the process of nitrification and denitrification slows down due to the decrease in temperature in the dry season. Generally speaking, in the natural environment, the removal capacity of TP through biological action only is low.

Overall, the four indicators of DO, COD_Mn_, COD_Cr_, and BOD indicated that the water quality in the dry season was better than that in the wet season, which were more affected by emissions. On the contrary, the two indicators of NH_4_^+^-N and TP in the wet season were better than in the dry season, which is more related to water volume. It can be considered that there was a more obvious tendency of organic pollution in the wet season, while the risk of nitrogen and phosphorus pollution was higher in the dry season in this area. Furthermore, S4 and S8 possessed more stable water quality in both seasons.

#### 3.1.3. Correlation of Indicators

**Figure 3:**
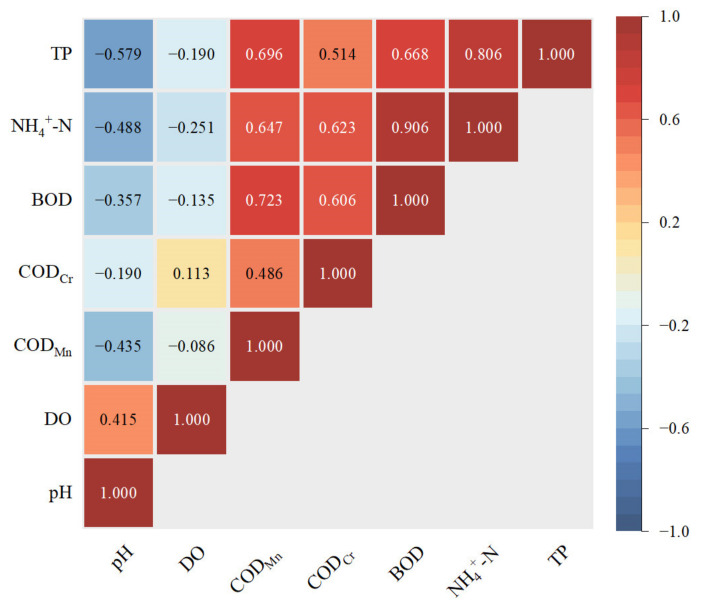
Correlation coefficients of annual water quality parameters.

In this section, the annual average value of each water quality parameter in each sampling site was calculated to obtain a total of 46 sets of water quality data. After that, a Pearson correlation analysis was conducted with annual data to reveal the correlation between every two water quality parameters of the basin. Figure 3 shows the correlation coefficient (cc) gradient according to color.

First, there was an obvious strong correlation between NH_4_^+^-N and BOD (cc = 0.906). BOD reflects the content of biodegradable organic matter in water. It can be inferred that the source of NH_4_^+^-N in the research area was highly related to the decomposition of biodegradable organics. There were also correlations between NH_4_^+^-N and COD_Mn_ (cc = 0.647), and between NH_4_^+^-N and COD_Cr_ (cc = 0.623), but the correlations were weaker than with BOD, which also supports this opinion.

There was also a strong correlation between NH_4_^+^-N and TP (cc = 0.806). The phenomenon of high correlation indicates that NH_4_^+^-N and TP usually showed consistent changes in water pollution. Nitrogen and phosphorus have similar sources, and both are important reasons for the eutrophication of water bodies. Conversely, eutrophication can lead to a further increase in NH_4_^+^-N.

BOD was correlated with COD_Mn_ and COD_Cr_, with cc = 0.723 and cc = 0.606, respectively. These three indicators can all represent the relative content of organic matter in the water body with different principles. BOD indicates some biodegradable organic matter in the water body, where the oxidant is dissolved oxygen. COD_Mn_ and COD_Cr_ can reflect the reducing substances in water, where the oxidants are potassium permanganate and potassium dichromate, respectively, with oxidation rates of about 40% and 90%, respectively. In accordance with the pollutants reflected in the three indicators, a correlation was anticipated, which was also confirmed statistically. The correlation coefficient shows that there was a higher correlation between the content of biodegradable organic matter and the content of substances that can be oxidized by potassium permanganate in this area.

In addition, there were also medial correlations between COD_Mn_ and TP (cc = 0.696), and between BOD and TP (cc = 0.668). It can be inferred that the TP was mainly composed of organic phosphorus compounds. The correlations among NH_4_^+^-N, TP, and organic indicators were consistent with the developed agricultural background in the research area.

### 3.2. Comprehensive Evaluation of Water Quality Based on WQI

In order to solve the bias and complexity of single-factor evaluation of water quality, a single value WQI was introduced to describe the overall water quality. According to the WQI classification standard, the evaluation results of 546 sets of monthly water quality data in the study period from 2013 to 2018 were “medium” (WQI ∈ [50–70)), “good” (WQI ∈ [70–90)), and “excellent” (WQI ∈ [90–100]), with no classifications of “poor” (WQI ∈ [25–50)) and “very poor” (WQI ∈ [0–25)).

The multiyear WQI of the whole region was 79.4, and the water quality was classified as “good”. During the study period, the multiyear water quality of each sampling site was evaluated as “good”. The sequence of multiyear WQI was S2 (73.9) < S6 (75.6) < S7 (76.1) < S4 (77.2) < S3 (79.4) = S5 (79.4) < S1 (85.0) < S8 (88.9). The WQI calculation results showed that the water quality of Xinchang River represented by S2 was the worst, followed by S6 of the mainstream of Cao’e River where the main tributaries converge. The water quality of S7 where the mainstream flows out of Shengzhou City ranked third. The water quality of S4 in the downstream flow was inferior to that of S3, upstream of Changle River. For Huangze River, the water quality of S5 was equivalent to that upstream of Changle River. The water quality of S1 on Chengtan River, the source of Cao’e River, was better, and that of S8 in Nanshan Reservoir was the best. Thus, the conclusion based on WQI was consistent with the water quality parameter analysis of Section 3.1.

On an annual timescale, the water quality classes of the whole region were “good”. The sequence of annual WQI values was 2013 (77.78) = 2014 (77.78) < 2015 (79.44) = 2016 (79.44) < 2018 (82.22) < 2017 (84.44). According to the evaluation results, the water quality showed a trend of gradual improvement. The water quality in 2013 was similar to that in 2014, whereas 2015 and 2016 were also similar. The water quality in 2017 was the best.

**Figure 4:**
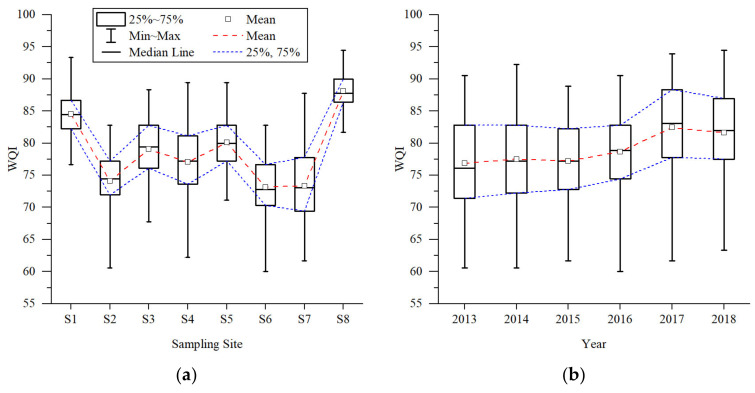
Box charts for WQIs of sampling sites (**a**) and study period (**b**).

The monthly WQI values of each sampling site and each year are drawn using two box charts to visually display the statistical characteristics of water quality, as shown in Figure 4. The box charts show the maximum and minimum values, upper and lower quartiles, and median and average values of each group of data.

It can be seen from the box chart of the sampling sites that the statistical law of monthly WQIs was consistent with the results of multiyear WQIs, although more information could be obtained. The water quality could be roughly divided into three groups according to the position and shape of the boxes: First, S8 with the best water quality and S1 with better water quality formed a group. Statistical characteristic values of WQI in S8 were the maximum, and the box was shortest, indicating that the WQI was relatively concentrated and the water quality was stable. The S1 box was shorter than other sites, and the water quality was stable. Second, S3, S4, and S5 with medium water quality were grouped together. Among the three sites, the median, average, and quartile of S4 were the smallest, indicating the water quality was poor. In addition, the span of the maximin was large and the box was long, meaning the water quality was unstable. The WQI boxes of S3 and S5 were similar in shape. The box position of S5 was slightly higher than that of S3, and the box was slightly shorter, indicating that the water quality was slightly better. Lastly, S2, S6, and S7 with worse water quality were grouped together. S2 had stable water quality, with the shortest box of the dataset. At the downstream site, the S7 box was long, indicating that the water quality changed greatly. According to all characteristic values, the water quality of S6 was the worst.

In the annual box chart, the statistical law of monthly WQI was consistent with the results of multiyear WQI. From 2013 to 2018, WQI generally showed a positive trend. The box shape in 2013 and 2014 was similar, but slightly better in 2014. In 2015 and 2016, the boxes became shorter, reflecting more stable water quality. The water quality was the best in 2017.

**Figure 5:**
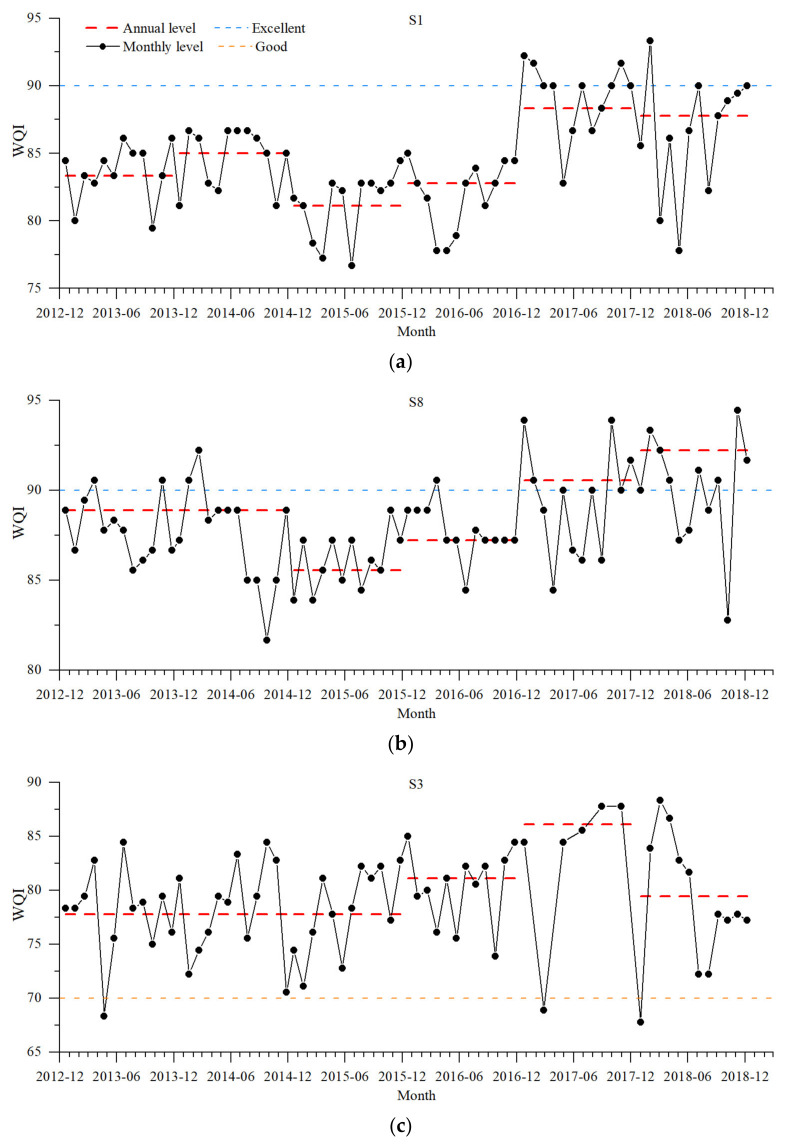

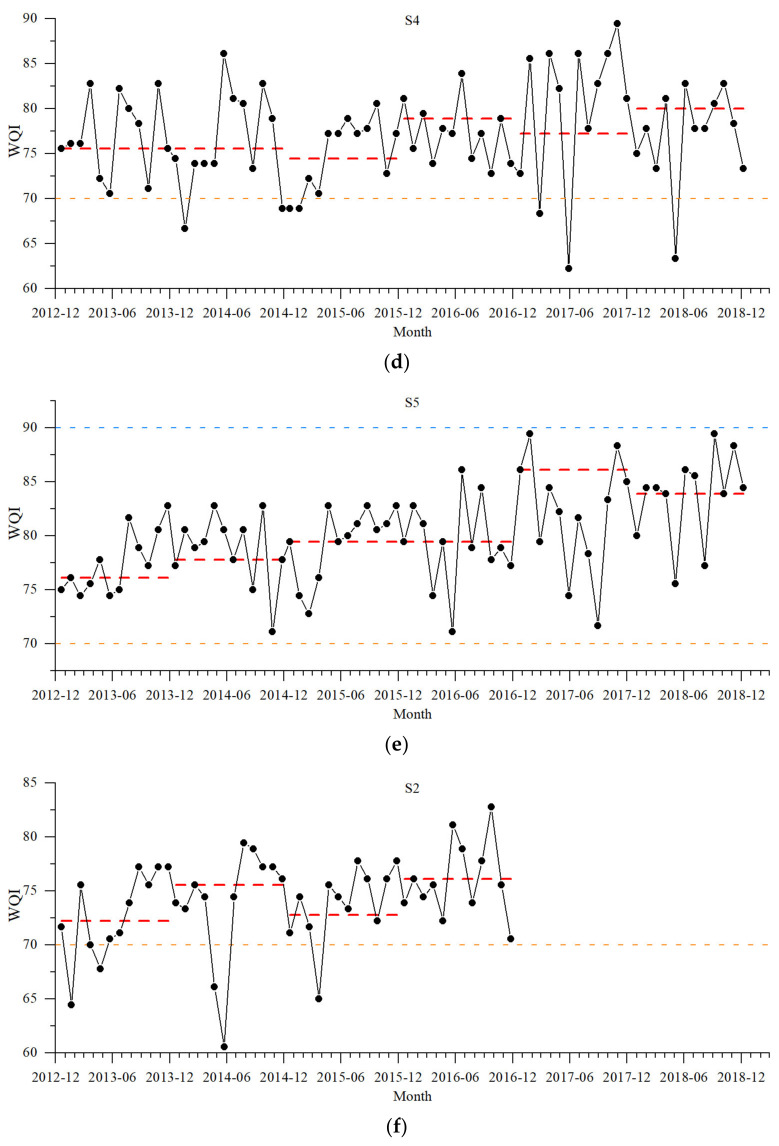

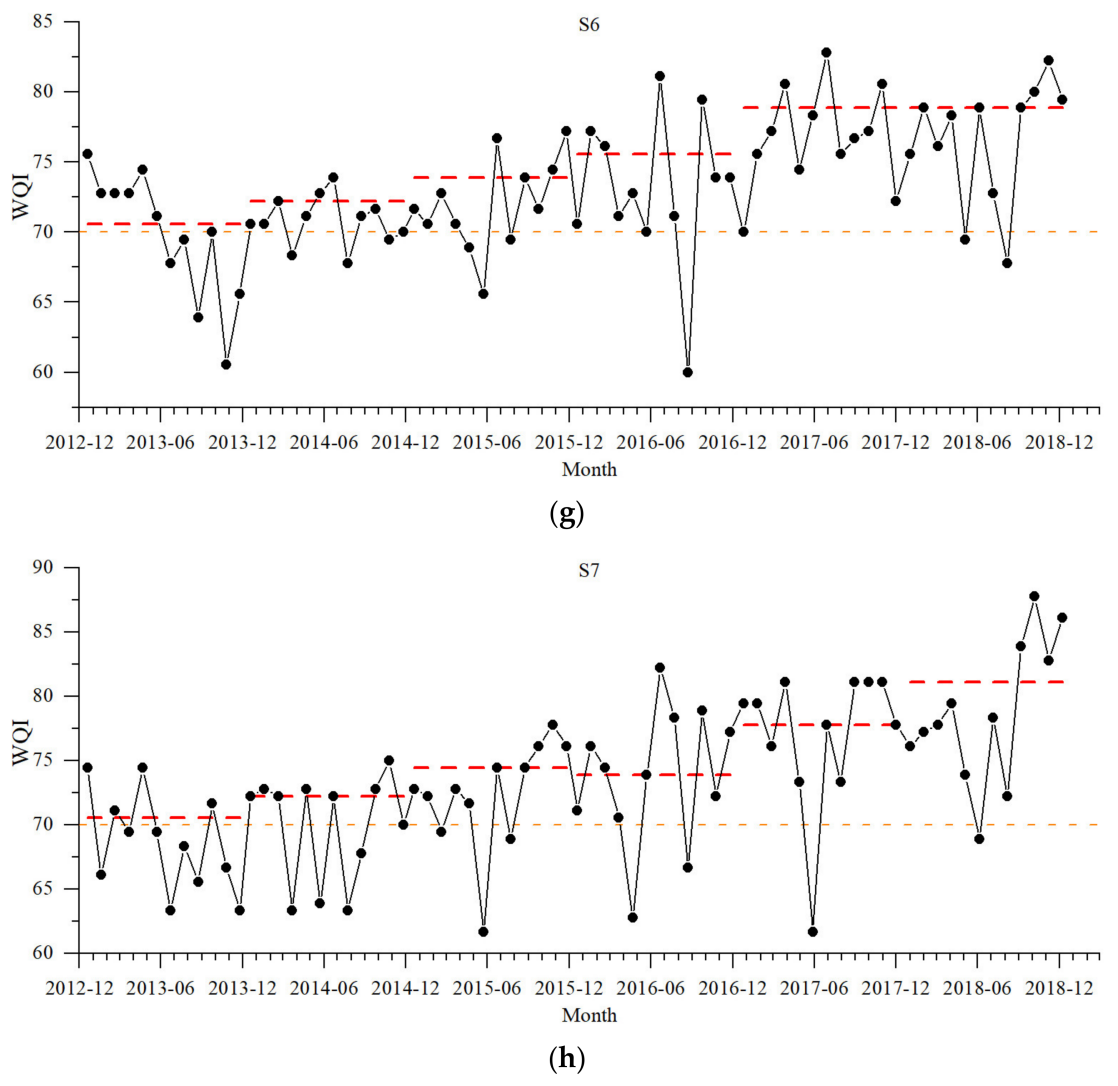
Monthly WQIs and annual WQIs of each site from 2013 to 2018.

The monthly WQIs and annual WQIs for each site are plotted as line graphs in Figure 5 to study the temporal variation of WQI for each site separately. On the basis of the grouping of the box charts above, the main findings are described below.

(1) S1 and S8:
  • The monthly WQIs were all high, with S1’s monthly WQIs above 75 and S8’s monthly WQIs all more than 80. Some monthly WQIs were even above 90, reaching the “excellent” standard.
  • The water quality in 2015 was the worst.
  • S1 had the best water quality in 2017, while S8 had the best water quality in 2018.
  • Water quality changed significantly during 2017 and 2018.

(2) S3, S4, and S5:
  • There were some monthly WQIs below 70 for S3 and S4, which belonged to the “medium” standard, while all values of S5 met the “good” standard. S3 had the worst water quality in January 2018, and S4 had the worst water quality in June 2017.
  • S4 had the worst water quality in 2015. S3 and S5 had the best water quality in 2017, while S4 had the best water quality in 2018.
  • The water quality changed the most during 2017.

(3) S2, S6, and S7:
  • There were more points with monthly WQIs less than 70 at the three sites, the water quality of which was “medium”. S2 had the lowest value in 2014-06, while the lowest value of S6 was in 2016-09, and that of S7 was in 2015-06 and 2017-06.
  • Distinguishing from the annual volatility of other sites, S6 and S7 had an obvious upward trend in annual WQIs, with the best water quality appearing in 2018.

Overall, combining the change characteristics of each site, three conclusions can be drawn. (1) The poorer water quality in 2015 and the higher water quality variability in 2017 require further study. (2) The fluctuation of water quality in tributaries during the year was greater than that of the mainstream, presenting a greater instability. (3) Low WQI points were more likely to occur in the wet season.

### 3.3. Spatial Distribution of Water Quality Based on CA

**Figure 6:**
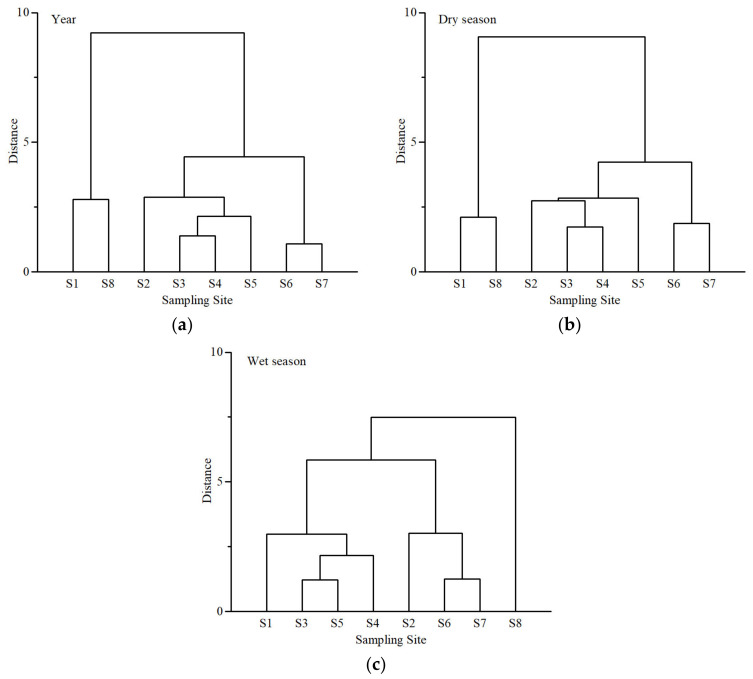
Clustering dendrograms of all years, as well as the dry and wet seasons. (**a**) Year; (**b**) Dry season; (**c**) Wet season.

The multiyear average and multiyear seasonal average water quality parameters of the eight sampling sites in the study area were categorized spatially on the basis of hierarchical cluster analysis, according to the comprehensive similarity of water quality parameters. Figure 6 includes three clustering dendrograms, displaying the water quality clustering results of all years, as well as the dry and wet seasons.

From a multiyear perspective, the eight sampling sites can be divided into three clusters: Cluster A (S1 and S8), Cluster B (S2, S3, S4, and S5), and Cluster C (S6 and S7). The multiyear water quality clustering results have obvious spatial location characteristics. Cluster A was located in the source location of the mainstream and the tributary. Cluster B includes the sampling points on all tributaries. Cluster C was located downstream of the study area. This shows that the sampling sites with the same location characteristics had similar water quality. In addition, Cluster A had the best water quality, followed by Cluster B, while the water quality of Cluster C was the worst. As seen from the dendrograms, the samples in Cluster C had the closest distance, indicating the highest similarity. The water quality parameters of S3 and S4 were the most similar in Cluster B, followed by S5, and finally S2. The samples in Cluster A had the least similarity. In terms of distance among clusters, Clusters B and C were closer and differed more from Cluster A.

The clustering result for the dry season was the same as that for the multiyear dataset. The distances among samples were closer. For the wet season, the eight sampling sites can also be divided into three distinct clusters: Cluster A (S8), Cluster B (S1, S3, S4, and S5), and Cluster C (S2, S6, and S7). Compared to all years and the dry season, the water quality of S3 and S5 was more similar in Cluster B in the wet season, followed by S4. Furthermore, S6 and S7 were closer in Cluster C.

### 3.4. Discrimination of Pollutants Based on PCA

#### 3.4.1. PCA of Research Area during the Period

This section attempts to identify control indicators and determine the main sources of pollution in water bodies according to the PCA of water quality parameters data. Depending on different data organization methods, multiple perspectives can be drawn. This section presents the PCA of the annual dry and wet seasons’ average values of water quality indicators for each site, including 46 sets of data, to identify the main pollutants of the research area and the difference between the seasons.

According to Section 3.1.2, there was a strong correlation between every two annual water quality parameters, indicating that there was a certain degree of overlap of water quality information. This makes the application of PCA suitable for dimensionality reduction, using fewer variables to reflect the water quality. The water quality parameters of the dry and wet seasons also had individually strong correlations which passed the applicability test.

The results of the PCA are presented in Table 4, which lists the names of the principal components, eigenvalues, percentage of variance explained, and cumulative contribution of the first three principal components for each period. The criteria of eigenvalues > 1 and the cumulative contribution rate close to 85% were used to screen the principal components.

On this basis, two principal components, PC1-Y and PC2-Y, were screened out from the PCA results of annual water quality, with eigenvalues of 4.032 and 1.298, and the percentage of variance explained was 57.601% and 18.546%, respectively. The cumulative contribution rate reached 76.147%, indicating that these two principal components reflect 76.147% of the original data information. Dry season water quality also yielded two principal components, PC1-D and PC2-D, with eigenvalues of 3.867 and 1.518, and contribution rates of 55.248% and 21.688%, respectively. The cumulative contribution rate was 76.936%, reflecting 76.936% of the information of the original data. Similarly, wet season water quality yielded two principal components, PC1-W and PC2-W, with eigenvalues of 3.741 and 1.104, and contribution rates of 53.438% and 15.773%, respectively. The cumulative contribution rate of the two principal components reached 69.211%.

Two principal components were separately identified for the data of annual water quality, and dry and wet season water quality, and further analyzed.

Table 5 presents the initial factor loading matrix, used to characterize the degree of correlation between the measured water quality indicators and the principal components.

A larger absolute value indicates a higher degree of correlation. The indicators with a high initial factor loading can be considered as the main control indicators to evaluate water quality. The principal component loading matrix (Table 6) presents the coefficients of water quality indicators on the principal components, which were obtained by dividing the initial factor loading matrix data by the square root of the corresponding eigenvalue of the principal component. Both the initial factor and the principal component loading matrixes can reflect the importance of the indicators to the principal components.

According to this principle, for PC1-Y of the annual water quality, explaining 57.601% of the variance, NH_4_^+^-N, BOD, TP, and COD_Mn_ had higher loadings. For PC2-Y, explaining 18.546% of the variance, DO had higher loading. For PC1-D of the dry season, explaining 55.248% of the variance, NH_4_^+^-N, BOD, COD_Mn_, and TP had higher loadings. For PC2-Y, explaining 21.688% of the variance, pH and DO had higher loadings. In summary, the PCA results of years and dry seasons were similar. NH_4_^+^-N, BOD, TP, and COD_Mn_ all had strong correlations with the first principal component, especially NH_4_^+^-N and BOD. The first principal component reflected that the water body was affected by nitrogen and phosphorus substances and organic matter nitrogen. In addition, the second principal component reflected the physical condition of the water body.

For PC1-W, explaining 53.438% of the variance in the wet season, NH_4_^+^-N and TP had higher loadings. For PC2-W, explaining 15.773% of the variance, DO had higher loading. The first principal component indicated that the wet season was mainly polluted by nitrogen and phosphorus, and the second principal component reflected the physical condition of the water body.

Combining these results, NH_4_^+^-N, BOD, TP, and COD_Mn_ can be used as control indicators for the watershed. It can be considered that the water environment in the research area was influenced by nitrogen and phosphorus substances and organic matter, especially biodegradable organic matter.

#### 3.4.2. PCA of Each Sampling Site and Each Year

PCA was performed for each sampling site and each year using the same method with measured monthly water quality data to determine the control water quality indicators. Key pollution indicators were defined as the top two water quality indicators ranked in the initial factor loading matrix and principal component loading matrix of the first principal components in the results of individual PCA. The results of each sampling site and each year are shown in Table 7.

One of the two key pollution indicators of all sampling sites was NH_4_^+^-N, indicating that nitrogenous substances were the main pollutants. In addition to NH_4_^+^-N, the key indicator of S1 was COD_Cr_, which was affected by reductive substances. Another key indicator of S2 and S6 was BOD, where the water quality was mainly affected by biodegradable organic substances. The key indicator of S3, S4, and S8 was COD_Mn_, indicating that the main pollutants of all sites in Changle River were reductive substances. For S5 with better water quality, the key indicator was DO, representing that the main influencing factor of S5 was the physical characteristics of the water environment. TP had a greater impact on S7.

For each year’s water quality, the key pollution factors in 2013, 2014, and 2016 were NH_4_^+^-N and TP, indicating that the water environment was mainly affected by nitrogen and phosphorus substances in these three years. For 2015, which had poor water quality, the key factors were COD_Cr_ and NH_4_^+^-N. It can be considered that the water quality was polluted by both reductive and nitrogenous substances. Organic matter was worthy of attention in 2017 as the key factors were COD_Mn_ and BOD, while the key factors of 2018 were COD_Mn_ and TP. With the improvement of water quality, the influence of NH_4_^+^-N tended to diminish.

## 4. Conclusions

This research used a combination of several research methods to reveal the water quality characteristics and the spatial and temporal changes of representative towns in the Yangtze River Delta region from different perspectives. The water quality characteristics of the study area are summarized below.

(1) The spatial variability of water quality was significant.

The water quality of S2, S4, S6, and S7 deserves further attention. The WQIs showed that the water quality of Xinchang River represented by S2 was the worst, followed by the water quality of S6 after the confluence of the main tributaries on the mainstream of Cao’e River, while the water quality of S7 near the location of the mainstream out of Shengzhou City was in third place. The water quality of S4 in the downstream position of Changle River was worse than that of S3 in the upstream position, and the water quality of S5 on Huangze River was at the same level as S3 upstream of Changle River. The water quality of Chengtan River, the source of Cao’e River, represented by S1, performed well, while S8 in Nanshan Reservoir was the best. Among the three tributaries, Xinchang River had the worst water quality, followed by Changle River, and Huangze River was the best. The water quality of the tributaries had higher volatility than the mainstream.

The eight sampling sites can be visually divided into three groups on the basis of the distribution pattern of the monthly WQI values. First, S8 and S1 with high stability and good water quality form a group. Second, S3, S4, and S5 with medium water quality can be grouped together. Lastly, S2, S6, and S7 with poor water quality make up the third group. According to the similarity of water quality indicators, the eight sampling points can be divided into three clusters using annual water quality data. Cluster A (S1 and S8) was located in the source location of the mainstream and tributary. Cluster B (S2, S3, S4, and S5) included the sampling sites on tributaries. Cluster C (S6 and S7) was located downstream of the study area. The sampling sites with similar locations had similar water quality characteristics.

(2) The water quality indicators differed in the dry and wet seasons.

For the four indicators of DO, COD_Mn_, COD_Cr_, and BOD, the water quality was better in the dry season than in the wet season. Conversely, for the two indicators of NH_4_^+^-N and TP, water quality was better in the wet season than in the dry season. This can be interpreted as a more obvious tendency of organic pollution in the wet season and a higher risk of nitrogen and phosphorus pollution in the dry season.

Low WQI points were more likely to appear in the wet season. According to the CA of sampling sites, there were also different clustering results in the dry and wet seasons.

(3) The overall water quality of the region improved over time.

The WQIs showed an improving trend in water quality, with the water quality condition being close in 2013 and 2014, similar in 2015 and 2016, with the best in 2017. Chengtan River, Xinchang River, and Changle River had worse water quality in 2015 than in 2014 and 2016, which was possibly related to the abundant precipitation and runoff of 2015. The key pollution factors of the study area in 2015 were COD_Cr_ and NH_4_^+^-N, and the water quality was affected by both reducing and nitrogenous substances. On the other hand, for 2017 with better water quality, the key factors were COD_Mn_ and BOD, and the water quality was mainly influenced by organic substances.

The main problems of the regional water environment are described below.

(1) Transboundary pollution was a significant problem.

With the developed water systems in Zhejiang Province, it is very common for rivers to span multiple districts, making it difficult to resolve the conflict of development and the environment between upstream and downstream. As a transboundary river, Xinchang River entered Shengzhou City from Xinchang County with poor water quality. Due to its location upstream of Cao’e River, it had a more serious impact downstream. The average COD_Cr_, BOD, NH_4_^+^-N, and TP of S2 were the largest among all sites, with the maximum records of COD_Mn_, BOD, and NH_4_^+^-N in the study area all appearing in S2.

The development of large chemical and pharmaceutical enterprises upstream has brought economic development to Xinchang County, while the discharge of enterprise sewage and wastewater applied serious pollution pressure to the water environment of Xinchang River.

(2) Nitrogenous substances and organic matter were the key pollutants.

NH_4_^+^-N, BOD, TP, and COD_Mn_ were the key water quality indicators in the study area. NH_4_^+^-N showed a significant impact on water quality at each sampling site, followed by organic matter-related indicators, with biodegradable organic matter in particular. Nitrogenous substances and organic matter were the main pollutants in the watershed. In addition, a strong correlation was shown between NH_4_^+^-N and BOD, along with a moderate correlation between NH_4_^+^-N and COD_Mn_. It can be presumed that ammonia nitrogen was mainly derived from the decomposition of organic nitrogen compounds, such as urea, amino acids, and proteins. The pollution pathways were agricultural fertilizers, human excretion in domestic sewage, and animal excretion from livestock farming.

(3) Non-point source pollution requires continuous control.

With the gradual completion of the urban drainage network in the study area, urban domestic sewage and industrial wastewater were effectively controlled. Therefore, non-point source pollution became an important source of pollution. There were intensive agricultural activities in the study area, with extensive sources of pollution from agricultural cultivation, livestock breeding, and rural living. In addition to the driving forces provided by regional precipitation and runoff characteristics, the risk of non-point source pollution was high. Water quality indicators differed in the dry and wet seasons. Along with abundant precipitation in the wet season, a large amount of organic matter entered the water body, which may have come from organic matter and chemical fertilizers in the soil, livestock and poultry excrement, and rural domestic sewage. Relevant remediation measures have been effective in recent years, and non-point source pollution control remains an important direction in coordinating industrial development and ecological environmental protection.

## Figures and Tables

**Table 1:** Score and weight of water quality parameters for the WQI method.

Parameter	Relative Weight (*P_i_*)	Normalization Factor (*C_i_*)										
		100	90	80	70	60	50	40	30	20	10	0
pH	1	7	7–8	7–8.5	7–9	6.5–7	6–9.5	5–10	4–11	3–12	2–13	1–14
DO	4	≥7.5	>7.0	>6.5	>6.0	>5.0	>4.0	>3.5	>3.0	>2.0	≥1.0	<1.0
COD_Mn_	3	<1	<2	<3	<4	<6	<8	<10	<12	<14	≤15	>15
COD_Cr_	3	<5	<10	<20	<30	<40	<50	<60	<80	<100	≤150	>150
BOD	3	<0.5	<2	<3	<4	<5	<6	<8	<10	<12	≤15	>15
NH_4_^+^-N	3	<0.01	<0.05	<0.10	<0.20	<0.30	<0.40	<0.50	<0.75	<1.00	≤1.25	>1.25
TN	3	<0.1	<0.2	<0.35	<0.5	<0.75	<0.10	<1.25	<1.50	<1.75	≤2	>2
TP	1	<0.01	<0.02	<0.05	<0.1	<0.15	<0.2	<0.25	<0.3	<0.35	≤0.4	>0.4

**Table 2:** Summary of the water quality parameters of research area.

Parameter	Units	n	Min	Max	Mean	SD
pH	Unitless	546	6.110	8.960	7.459	0.424
DO	mg/L	546	3.120	15.500	8.737	1.615
COD_Mn_	mg/L	546	0.080	7.430	2.693	0.802
COD_Cr_	mg/L	546	2.000	28.000	11.273	3.003
BOD	mg/L	546	0.250	6.510	2.315	1.042
NH_4_^+^-N	mg/L	546	0.007	1.720	0.374	0.286
TP	mg/L	546	0.003	0.289	0.077	0.044

**Table 3:** Water quality characteristics in different sampling sites.

Site	Sample Number	pH		DO		COD_Mn_		COD_Cr_		BOD		NH_4_^+^-N		TP	
		Mean	SD	Mean	SD	Mean	SD	Mean	SD	Mean	SD	Mean	SD	Mean	SD
S1	72	7.487	0.417	9.279	1.446	2.213	0.575	10.486	2.892	1.703	0.783	0.169	0.116	0.049	0.025
S2	48	7.434	0.377	9.196	1.187	2.738	0.824	12.458	1.798	3.017	1.003	0.671	0.286	0.113	0.049
S3	66	7.407	0.365	9.109	1.475	2.974	0.833	11.833	3.036	2.156	1.026	0.340	0.200	0.080	0.036
S4	72	7.490	0.395	9.287	1.955	3.179	0.864	12.271	3.099	2.510	1.039	0.398	0.273	0.106	0.037
S5	72	7.386	0.295	8.563	1.404	2.549	0.531	11.382	2.954	2.072	0.799	0.321	0.209	0.080	0.034
S6	72	7.320	0.362	7.871	1.490	2.928	0.598	11.196	2.531	2.941	0.844	0.605	0.232	0.103	0.024
S7	72	7.363	0.328	7.746	1.671	2.997	0.767	12.042	3.367	2.835	1.079	0.515	0.277	0.087	0.036
S8	72	7.770	0.609	9.030	1.217	2.008	0.600	8.958	2.328	1.509	0.593	0.068	0.042	0.014	0.005

**Table 4:** Eigenvalue and principal component contribution rate.

Period	Principal Component Number	Eigenvalue	Percentage of Variance (%)	Cumulative (%)
Year	PC1-Y	4.032	57.601	57.601
	PC2-Y	1.298	18.546	76.147
	PC3-Y	0.581	8.304	84.450
Dry season	PC1-D	3.867	55.248	55.248
	PC2-D	1.518	21.688	76.936
	PC3-D	0.697	9.954	86.890
Wet season	PC1-W	3.741	53.438	53.438
	PC2-W	1.104	15.773	69.211
	PC3-W	0.756	10.804	80.015

**Table 5:** Initial factor loading matrix.

Water Quality Parameter	Year		Dry Season		Wet Season	
	PC1-Y	PC2-Y	PC1-D	PC2-D	PC1-W	PC2-W
pH	−0.620	0.551	0.059	0.796	−0.785	0.399
DO	−0.255	0.846	−0.077	0.794	−0.379	0.855
COD_Mn_	0.820	0.110	0.890	0.032	0.703	0.233
COD_Cr_	0.681	0.482	0.775	0.427	0.611	0.121
BOD	0.890	0.177	0.913	−0.013	0.797	0.361
NH_4_^+^-N	0.936	0.029	0.933	−0.168	0.888	0.121
TP	0.884	−0.051	0.873	−0.204	0.831	0.006

**Table 6:** Principal component loading matrix.

Water Quality Parameter	Year		Dry Season		Wet Season	
	PC1-Y	PC2-Y	PC1-D	PC2-D	PC1-W	PC2-W
pH	−0.308	0.483	0.030	0.646	−0.406	0.380
DO	−0.127	0.743	−0.039	0.644	−0.195	0.814
COD_Mn_	0.408	0.096	0.453	0.026	0.363	0.221
COD_Cr_	0.339	0.422	0.394	0.347	0.316	0.114
BOD	0.443	0.155	0.464	−0.010	0.412	0.343
NH_4_^+^-N	0.466	0.025	0.474	−0.136	0.459	0.115
TP	0.440	−0.045	0.444	−0.166	0.430	0.007

**Table 7:** Key pollution indicators of each sampling site and each year.

Sampling Site	Pollution Factors		Year	Pollution Factors	
S1	COD_Cr_	NH_4_^+^-N	2013	NH_4_^+^-N	TP
S2	NH_4_^+^-N	BOD	2014	NH_4_^+^-N	TP
S3	COD_Mn_	NH_4_^+^-N	2015	COD_Cr_	NH_4_^+^-N
S4	COD_Mn_	NH_4_^+^-N	2016	NH_4_^+^-N	TP
S5	NH_4_^+^-N	DO	2017	COD_Mn_	BOD
S6	BOD	NH_4_^+^-N	2018	COD_Mn_	TP
S7	COD_Mn_	TP			
S8	COD_Mn_	NH_4_^+^-N			

## Data Availability

Not applicable.
